# Polymorphisms of Mismatch Repair Pathway Genes Predict Clinical Outcomes in Oral Squamous Cell Carcinoma Patients Receiving Adjuvant Concurrent Chemoradiotherapy

**DOI:** 10.3390/cancers11050598

**Published:** 2019-04-29

**Authors:** Thomas Senghore, Wen-Chang Wang, Huei-Tzu Chien, You-Xin Chen, Chi-Kuang Young, Shiang-Fu Huang, Chih-Ching Yeh

**Affiliations:** 1 School of Public Health, College of Public Health, Taipei Medical University, Taipei 11031, Taiwan; tsenghore@gmail.com (T.S.); youxin810@gmail.com (Y.-X.C.); 2 Department of Nursing, School of Medicine and Allied Health Sciences, University of The Gambia, Independence Drive, Banjul, P. O. Box 1646, The Gambia; 3 Ph.D. Program for Translational Medicine, College of Medical Science and Technology, Taipei Medical University, Taipei 11301, Taiwan; wangwc@tmu.edu.tw; 4 Department of Public Health, Chang Gung University, Taoyuan 33305, Taiwan; kathy.htchien@gmail.com; 5 Department of Nutrition and Health Sciences, Chang Gung University of Science and Technology, Taoyuan 33302, Taiwan; 6 Department of Otolaryngology, Chang Gung Memorial Hospital, Keelung 20401, Taiwan; rioriorioman@gmail.com; 7 Department of Otolaryngology, Head and Neck Surgery, Chang Gung Memorial Hospital, Linkou, Taoyuan 33305, Taiwan; 8 School of Public Health, College of Public Health, China Medical University, Taichung 40402, Taiwan

**Keywords:** mismatch repair gene, oral squamous cell carcinoma, single-nucleotide polymorphism, concurrent chemoradiotherapy, clinical outcome

## Abstract

Background: We aimed to investigate the association between single-nucleotide polymorphisms (SNP) in mismatch repair (MMR) pathway genes and survival in patients with oral squamous cell carcinoma (OSCC) who received adjuvant concurrent chemoradiotherapy (CCRT). Methods: Using the Sequenom iPLEX MassARRAY system, five SNPs in four major MMR genes were genotyped in 319 patients with OSCC who received CCRT treatment. Kaplan–Meier survival curves and Cox proportional hazard regression models were used to assess overall survival (OS) and disease-free survival (DFS) among MMR genotypes. Results: The results of Kaplan–Meier survival analysis revealed that the MutS homolog 2 *(MSH2)* rs3732183 polymorphism showed a borderline significant association with DFS (log-rank *p* = 0.089). Participants with the *MSH2* rs3732183 GG genotype exhibited a relatively low risk of recurrence (hazard ratio (HR) = 0.45; 95% confidence interval (CI) = 0.22–0.96; *p* = 0.039). In addition, the MutL homolog 1 (*MLH1)* rs1800734 GG genotype carriers exhibited higher OS (HR = 0.52, 95% CI = 0.27–1.01; *p* = 0.054) and DFS (HR = 0.49, 95% CI = 0.26–0.92; *p* = 0.028) rates. Conclusions: Our results indicated that the GG genotypes of *MSH2* rs3732183 and *MLH1* rs1800734 are associated with relatively high survival in OSCC patients treated using adjuvant CCRT. These polymorphisms may serve as prognosis predictors in OSCC patients.

## 1. Introduction

Approximately 3% of all cancers occur in the oral cavity; approximately 90% of all oral cancers (OCs) are oral squamous cell carcinoma (OSCC), which is one of the most prevalent cancers and the fourth commonest causes of cancer mortality among men in Taiwan [1]. Despite advancements in the diagnosis and treatment of OSCC, the 5-year overall survival (OS) rate, which is currently 56.6%, has not improved significantly in the past decades [1]. This is mainly because approximately 51.5% of OSCC cases are diagnosed at advanced stages (III and IV) [1]. For the early stages, namely stages I and II, surgery constitutes the standard treatment of choice and often results in a permanent cure. Adjuvant treatments of chemotherapy and radiotherapy (RT) with surgery are used to treat advanced stages of OSCC, depending on the overall health status of the patients and invasive pathological parameters [2,3]. Despite aggressive therapy, the 5-year survival rate among patients with advanced stages of OSCC is low, and most patients die in the first 30 months of the disease [4,5]. The clinical outcomes of OSCC often vary with factors related to the tumor and treatment [6]. However, inherent patient genetic characteristics may affect patient prognosis [7].

The response of tumor cells to DNA damage involves numerous complicated molecular mechanisms [8,9]. For example, DNA repair mechanisms can affect the survival of tumor cells. Both chemotherapy and RT induce DNA lesions, including replication errors, thereby activating the DNA damage and repair response [10]. The DNA mismatch repair (MMR) pathway proteins, which include MutL homolog 1 (MLH1), MutS homolog 2 (MSH2), MutS homolog 3 (MSH3), and exonuclease 1 (EXO1), play significant roles in the repair process by recognizing DNA damage caused by endogenous and exogenous agents [11,12]. MSH2, which dimerizes with other proteins (MSH6 or MSH3), and are responsible for recognizing and initiating the mismatch repair. The MLH1 protein joins another protein (MLH4) to form a protein complex that coordinates downstream repair events, which involves the EXO1 protein [12]. Genetic variations in MMR genes alter the capacity of individuals to repair DNA damage induced by radiotherapeutic and chemotherapeutic agents, leading to variations in outcomes ranging from tumor cell apoptosis to resistance [13,14]. For example, the G-alleles of *MLH1* rs1800734 [15], *MSH3* rs26279 [14,16], and *EXO1* rs1047840 [14] have been reported to be associated with higher levels of protein expression and/or higher survival rates than the variant alleles. Thus, the G-alleles represent higher DNA repair capacity than the other alleles, indicating that these single-nucleotide polymorphisms (SNPs) may have the potential to become prognostic biomarkers for individualized therapy.

Studies on the effects of genetic variations in MMR genes on the prognosis of cancer have generally included patients receiving different treatments and/or patients with different ethnic backgrounds [14,16,17,18]. Given that different treatments may cause varying DNA damage and exhibit different repair efficacy, we investigated the association between polymorphisms and outcomes in OSCC patients who received identical treatment with adjuvant concurrent chemoradiotherapy (CCRT) and exhibited similar disease stages. Five SNPs of *MSH2*, *MSH3*, *EXO1,* and *MLH1* were selected according to their effects on the risk and/or survival of tumors [14,16,19,20].

## 2. Results

In this study, 319 male patients with OSCC were recruited to explore the effects of genetic variants of MMR genes on the risk of death or recurrence. Their demographic and clinical parameters were evaluated (Table 1). The mean (± standard deviation) age of the participants was 49.72 (± 9.8) years, and approximately half (48.59%) of the patients were ≥50 years old. Most of the participants were of Taiwanese ethnicity (72.1%), had normal body mass index (BMI) (49.22%), and reported that they had ever smoked cigarettes (85.27%), drank alcohol (69.28%), and chewed betel quid (86.21%). The participants who exhibited poor tumor characteristics comprised 17.24% with poor tumor differentiation, 61.76% with a primary tumor size corresponding to the T3–T4 range, 55.49% with perineural invasion, 5.96% with vascular invasion, 12.54% with lymphatic invasion, 64.26% with extranodal extension (ENE), and 86.83% with pathological tumor, nodes, and metastasis (TNM) stage IV.

The maximum follow-up period was 199 months with median follow-up times of 16 and 13 months for OS and disease-free survival (DFS), respectively. For the entire follow-up duration, 94 patients (29.5%) and 129 patients (40.4%) experienced events of death and recurrence, respectively. In the univariate analysis, clinicopathological characteristics, such as nodal involvement in the N2–N3 range (hazard ratio (HR) = 2.38, 95% confidence intervals (CI) = 1.43–3.94; *p* = 0.0008), lymphatic invasion (HR = 2.22, 95% CI = 1.32–3.72; *p* = 0.003), and ENE (HR = 3.78, 95% CI = 2.14–6.68; *p* < 0.001), were associated with poor OS, and primary tumor size in the T3–T4 range (HR = 1.47, 95% CI = 1.03–2.09; *p* = 0.034), nodal involvement in the N2–N3 range (HR = 1.76, 95% CI = 1.17–2.63; *p* = 0.006), and ENE (HR = 1.87, 95% CI = 1.26–2.78; *p* = 0.002) were associated with poor DFS (Table 2).

Furthermore, univariate candidate SNP analysis revealed that *MSH2* rs3732183 GG genotype (codominant model: HR = 0.47, 95% CI = 0.22–0.97; *p* = 0.042 and recessive model: HR = 0.46, 95% CI = 0.22–0.94; *p* = 0.034) was a more favorable prognostic factor for predicting DFS than was the AA genotype. The *EXO1* rs1047840 AA (recessive model: HR = 2.80, 95% CI = 1.02–7.66; *p* = 0.045) genotype was associated with a significantly increased risk of recurrence than the wild-type genotype (Table 3). In addition, the *MLH1* rs1800734 GG genotype (codominant model: HR = 0.59, 95% CI = 0.33–1.06; *p* = 0.077) was associated with a slightly but insignificantly lower risk of DFS than the AA genotype. Although the *MSH3* polymorphisms were not significantly associated with survival, the linkage disequilibrium (LD) analysis show that *MSH3* rs12515548 and rs26279 were in high LD with each other (D’ = 0.97 and R^2^ = 0.94) (Figure S1). Therefore, only the missense SNP (rs26279) was used in further analysis.

**Figure 1:**
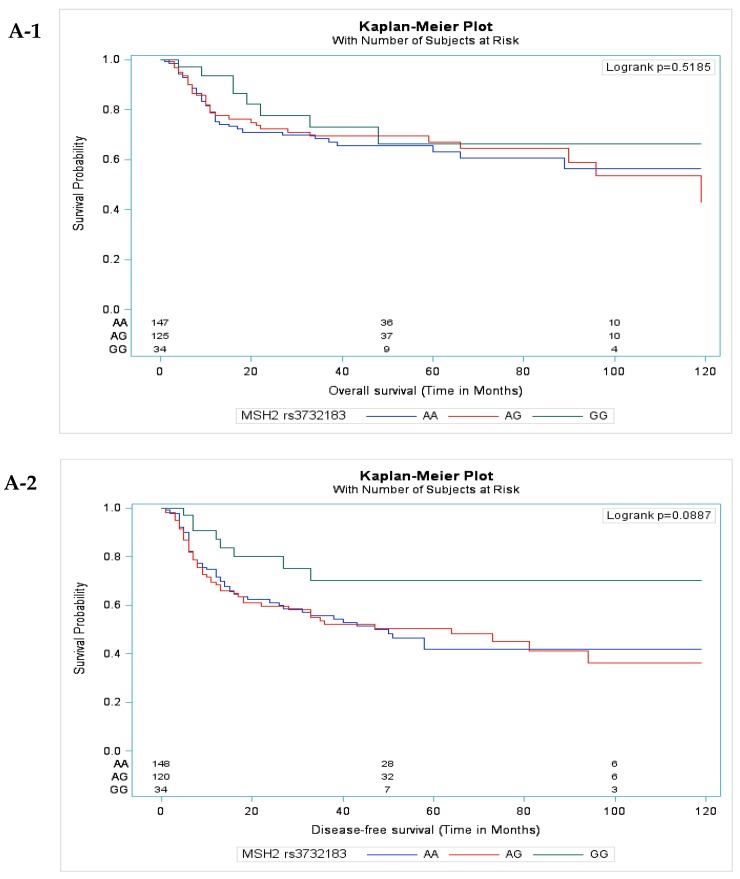

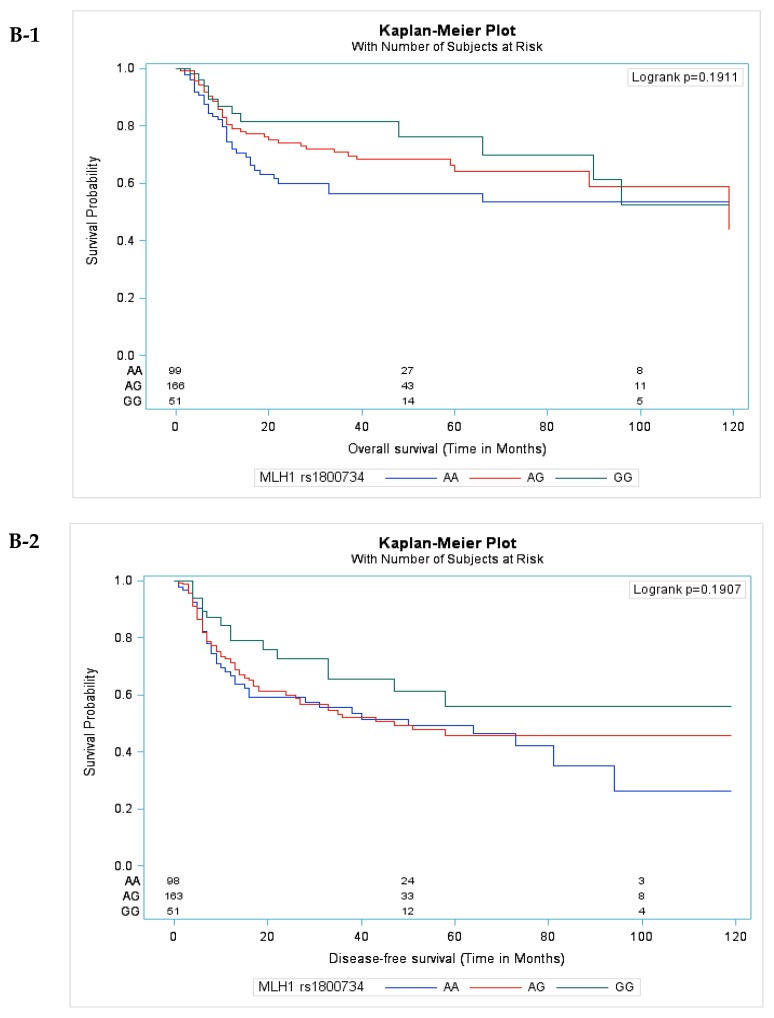
Kaplan–Meier analysis and log-rank test results of OS and DFS curves for the *MSH2* rs3732183 and *MLH1* rs1800734 polymorphisms in patients with oral squamous cell carcinoma treated with concurrent chemoradiotherapy. (**A-1**) No significant difference in OS among the *MSH2* rs3732183 genotypes (log-rank test *p =* 0.5185). (**A-2**) The GG genotype showed a borderline significant better DFS time than the AA genotype (log-rank test *p =* 0.0887). (**B-1,2**) No significant difference in death and recurrence among genotypes of *MLH1* rs1800734 (log-rank test *p =* 0.1911 and *p =* 0.1907, respectively).

Figure 1 shows the Kaplan–Meier analysis and log-rank test results of the OS and DFS curves for selected SNPs in the OSCC patients who received CCRT. A borderline significant difference was observed in the DFS time but not in the OS time among the *MSH2* rs3732183 genotypes. The individuals with the GG genotype exhibited better DFS time than the individuals with other genotypes (log-rank test *p* = 0.0887) (Figure 1 (A-1,A-2)). However, no significant difference in OS time was observed among the individuals with the *MLH1* rs1800734 genotypes (Figure 1 (B-1,B-2)).

Table 4 shows the final multivariable model including demographic, clinical, and genetic factors for OS and DFS, respectively. The presence of ENE (HR = 2.91, 95% CI = 1.58–5.34, *p* = 0.006) was associated with a relatively high mortality risk. Patients of Mainland Chinese ethnicity (HR = 1.99, 95% CI = 1.04–3.82; *p* = 0.039), primary tumor size in the T3–T4 range (HR = 1.88, 95% CI = 1.19–2.98; *p* = 0.007), and nodal involvement in the N2–N3 range (HR = 1.96, 95% CI = 1.04–3.69; *p* = 0.038) were associated with an increased risk of recurrence. The GG genotype of *MLH1* rs1800734 was associated with borderline statistically significant longer OS (codominant model: HR = 0.52, 95% CI = 0.27–1.01; *p* = 0.054). The GG genotypes of *MSH2* rs3732183 (codominant model: HR = 0.45, 95% CI = 0.22–0.96; *p* = 0.039) and *MLH1* rs1800734 (codominant model: HR = 0.49, 95% CI = 0.26–0.92; *p* = 0.028) were favorable prognostic predictors of DFS.

## 3. Discussion

The MMR is a complicated network with numerous functions. One prominent function is genomic stability, which is achieved by eliminating mismatched or distorted DNA strands. SNPs in this pathway are shown to alter the anticancer effects of therapeutic agents. Therefore, identifying such SNPs may determine prognostic markers in patients with OSCC. In this retrospective cohort study, we investigated the association between genetic variants of MMR genes and clinical outcomes in the patients with OSCC who received adjuvant CCRT. The results indicated that the *MSH2* rs3732183 GG genotype was a favorable prognostic indicator of relapse, and the *MLH1* rs1800734 GG genotype was a favorable prognostic indicator of both relapse and death. In addition, patients with ENE exhibited a high risk of death, while those of Mainland Chinese ethnicity, with primary tumor size in the T3–T4 range and nodal involvement in the N2–N3 range exhibited high risks of relapse.

*MSH2* is a prominent member of the MMR pathway, and its inactivation can have far-reaching pathological effects on DNA. Studies have reported that polymorphisms in this gene affect the DNA damage and repair mechanism [14,21]. Similarly, we found that variations in *MSH2* affected clinical outcomes in patients with OSCC. Patients with the rs3732183 GG genotype exhibited a lower risk of relapse than the patients with the AA genotype. The SNP rs3732183 is an intronic SNP that may affect MSH2 expression through cis-acting regulatory elements (such as enhancers, silencers, insulators, and transcription factors) that positively control gene expression [22]. The A to G substitution may result in high MSH2 expression, which is favorable to the anticancer effects of therapeutic agents, thus reducing the risk of relapse. Low expression levels of MSH2 were previously found to have unfavorable prognostic value for different cancers, including head and neck squamous cell carcinoma (HNSCC) [18,23,24]. Huang et al. reported MSH2 overexpression to correlate with better survival in Taiwanese colon cancer patients [25]. Alternatively, the SNP may be in high linkage disequilibrium with other nearby polymorphisms that may affect MSH2 expression. Previously, Kang et al., in their study on Korean colorectal cancer patients, also reported the GG genotype of rs3732183 to show a favorable prognostic factor for DFS [26].

Consistent with the results of previous studies [7,27], we also observed that the carriers of the *MLH1* rs1800734 GG genotype exhibited lower risks of relapse and death than did the carriers of the wild-type AA genotype. The SNP rs1800734 is located at an *MLH1* promoter CpG island transcription factor-binding site that can cause differences in individual susceptibility by regulating the activity of MLH1 and other downstream proteins [28]. Several studies have reported that rs1800734 is associated with high levels of methylation and low levels of protein expression in different types of tumors [29,30,31], which suggests that this polymorphism may contribute to gene dysfunction by altering transcription activity. The GG genotype carriers may be at a relatively low risk because the G-allele provides a favorable binding site for transcription factors, such as AP-4, which recruits RNA polymerase II and c-Myc, thereby leading to *MHL1* transcription [28]. By contrast, a repressor protein may bind to the A-allele to recruit epigenetic modifying factors leading to promoter methylation and low level of *MHL1* transcription [29]. Extensive promoter methylation was associated with *MLH1* inactivation [32]. The AA genotype of *MLH1* rs1800734 was previously found to be correlated with poor prognosis in Taiwanese patients with lung cancer [27]. Our findings support the proposition that reduced DNA damage efficacy offers a survival advantage in patients with OSCC.

A few studies have reported relatively poor survival outcomes in the *EXO1* rs1047840 and *MSH3* rs26279 GG genotypes among patients with HNSCC [14,16]. Nogueira et al. reported that the GG genotypes of *EXO1* rs1047840 and *MSH3* rs26279 were associated with DFS and OS, respectively, in a Brazilian population with HNSCC. A similar Brazilian study on patients with HNSCC showed that the *MSH3* rs26279 GA or AA genotypes are associated with an approximate nine-fold higher risk of partial response to cisplatin chemoradiation or achieving stable disease than are patients with other genotypes [14]. The *EXO1* rs1047840 GA genotype was associated with complete recovery in patients with laryngeal cancer treated using cisplatin and RT [33]. However, we did not observe any association between the SNPs and clinical outcomes in our sample. The difference between the results of the previous study and those of the present study may be attributable to differences in ethnicity, tumor characteristics, treatment, and median follow-up times. Our sample was highly homogeneous and included patients with advanced OSCC of identical ethnicity who received identical treatment, whereas the study samples in the aforementioned studies consisted of patients with diverse tumor characteristics who received different treatment modalities. In addition, despite the long follow-up time of 199 months in our study, the median follow-up time in this study was shorter than that in other studies [14,16] and could be attributed to the poor tumor characteristics of our patients. The difference in findings may also be attributed to the low statistical power of our study. Additional studies with larger samples than that in the present study may be required to confirm the results of the present study.

As previously reported [14,16,34], we observed that the participants with ENE exhibited an increased risk of death and those with primary tumor size in the T3–T4 range and nodal involvement exhibited poorer DFS than their counterpart with milder characteristics. Epidemiological studies from Taiwan showed ethnic difference in oral cancer survival [35,36]. Similarly, in our study mainland Chinese participants exhibited increased risk for recurrence than does participants of Taiwanese ethnicity.

The strength of our study is the relative homogeneity of our patient population with respect to ethnicity, treatment modality, and tumor stage, which eliminated the effects of differences in DNA damage and repair mechanisms and their effects on clinical outcomes. The relatively small sample owing to the strict inclusion criteria and the recruitment of patients from only one hospital may have limited the generalizability of our findings. Despite the aforementioned limitation, this is the first study to investigate the association between genetic polymorphisms in MMR genes and clinical outcomes in patients of Chinese ethnicity with advanced OSCC receiving adjuvant CCRT.

## 4. Materials and Methods

### 4.1. Ethical Statement

The study was conducted in accordance with the Declaration of Helsinki. The ethics review committees of the Chang Gung Memorial Hospital (IRB No. 201800213B0) and the Taipei Medical University (IRB No. N201802083) granted final approval for the study. Written informed consent to participate in the study was obtained from all the participants after a detailed explanation of the study objectives.

### 4.2. Participants and Data Collection

For this study, participants were recruited from the Chang Gung Memorial Hospital, Linkou, Taiwan, and constituted a part of the Head and Neck Surgery Department’s Cancer Registry from 1999 to 2016. The registry included 2528 cases of OSCC. After excluding 51 cases who had incomplete questionnaires, there were 973 cases who received surgery only, 513 cases who had surgery plus radiotherapy, 503 cases who had post-operative CCRT, and 488 cases who received other treatment or had missing information on treatment. We selected those patients who had received CCRT and restricted the sample to male subjects, which left a sample of 473 for the present study. Patients were further excluded if they had no blood specimen (*n* = 105), were of aboriginal ethnicity (*n* = 13), had early-stage OSCC (*n* = 12), had missing clinicopathological information (*n* = 12), or had failed the genotyping experiment (*n* = 7). Finally, 319 participants were included in the analysis. The sociodemographic data and lifestyle habits of the participants were obtained using an interviewer-administered questionnaire. The collected information included age, education level, ethnicity, lifestyle habits (history of alcohol drinking, betel quid chewing, and cigarette smoking), and family history of cancers. Lifestyle habits were dichotomized into never (never engaged in the habit for over 1 year continuously) and ever (had ever engaged in the habit for over 1 year continuously). Detailed clinical information was also collected by taking medical history, evaluating complete blood count, and conducting complete physical examination, routine blood chemistry tests, whole-body bone scan or positron emission tomography scan, abdominal ultrasound and magnetic resonance imaging (MRI), or computed tomography (CT) of the head and neck. The clinicopathological characteristics of the patients included tumor stage, depth, and differentiation, nodal status, and ENE, as well as perineural, skin, and bone invasions. The participants’ weight and height were also recorded, and BMI was computed using the following formula: weight/height^2^ (kg/m^2^). 

### 4.3. DNA Extraction and Genotyping

Pairs of samples from the tumor and normal adjacent nontumor tissue that were previously obtained from each participant and stored in liquid nitrogen (at −80 °C) were used for pathological examination and staging by a pathologist. The Seventh Edition of the American Joint Committee on Cancer (AJCC)–TNM staging system [37] was used for staging. Only participants diagnosed with histological squamous cell carcinoma and TNM stages III and IV were considered for the study. Prior to treatment, the participants’ venous blood samples were collected and centrifuged, and buffer-coated cells were collected for DNA extraction. Genomic DNA extraction was performed using the standard phenol–chloroform method.

Extracted DNA was used for genotyping the *MSH2* SNP rs3732183, *MSH3* SNPs rs12515548 and rs26279, *EXO1* SNP rs1047840, and *MLH1* SNP rs1800734, which were selected based on their previously reported association with the risk or prognosis of cancers [14,16,19,20]. The Sequenom iPLEX MassARRAY system (Sequenom, Inc., San Diego, CA, USA) was used for genotyping. Using the Sequenom MassARRAY platform and iPLEX GOLD chemistry, matrix-assisted laser desorption ionization time of flight (MALDI-TOF) spectroscopy was conducted. Then, 10 ng of genomic DNA was used as a template, and a polymerase chain reaction (PCR) mixture containing Qiagen HotStarTaq (Qiagen, Valencia, CA, USA) was prepared. Primer extension and cleanup using shrimp alkaline phosphatase were performed according to the Sequenom guidelines. The PCR primers were obtained from Integrated DNA Technology (Coralville, IA, USA). The MassARRAY Assay Design software Version 3.1 (Sequenom) was used for assay design. For quality control, 10% of samples were randomly selected, and genotypes showed 100% concordance for all SNPs.

### 4.4. Adjuvant CCRT and Follow-Up

Prior to CCRT treatment, all the patients had undergone surgery that included radical tumor excision with neck dissection based on clinical stage after tumor survey. The primary tumor resection was performed at 1 cm above safety margins (both peripheral and deep margins), and neck dissection was performed according to examination status. All the patients received CCRT with radiation doses between 6000 and 6600 cGy, bi-weekly or tri-weekly intravenous cisplatin 40–60 m^2^/kg, and oral 5-fluorouracil for 4–8 weeks after the surgical procedure. The patients were evaluated during and after treatment through regular clinical and radiological examinations. The examinations involved check-ups every month for the first 6 months, every 2 months for the next 6 months, every 3 months within the second year, and every 6 months thereafter. Monitoring included analysis of medical history and physical examination (including complete oral examination), X-rays, CT or MRI, and laboratory examination. History of biopsy or imaging studies were used to confirm relapse, and deaths resulting from OSCC were recorded based on death certificates.

### 4.5. Statistical Analysis

The major clinical outcome was relapse (DFS) of OSCC or death (OS) caused by OSCC. The DFS time (in months) was calculated from the day of treatment commencement to the time of relapse, metastasis, or death from any cause. The OS time was defined as the duration (in months) between the time of treatment commencement and date of death. The patients who did not experience any event as of the date of the last follow-up visit were censored or subject to administrative censoring by the end of the study period (10 April 2019). The demographic and clinicopathological parameters of the participants were summarized using descriptive statistics. Cumulative survival in different genotypes was analyzed using Kaplan–Meier curves, and the differences between the genotypes were tested using the log-rank test. The association among the demographic and clinicopathological parameters, candidate SNPs, and OS or DFS were tested using univariate and multivariate Cox proportional hazard regression models. Multivariate analysis was conducted to examine the associations between individual SNPs and OS or DFS after adjusting for age and clinicopathological variables that were significant at *p* < 0.10 in the univariate model. Different genetic models were used to test the effect of individual SNPs on OS and DFS (including codominant, additive, dominant, recessive, and allelic models). The most significant genetic model was finally used in the multivariate analysis. The relative risk of relapse or death was estimated using HR and their corresponding 95% CIs. For the two SNPs located in *MSH3*, LD analysis was performed using Haploview (version 4.2, Broad Institute, Cambridge, MA, USA). All analyses were two-sided, and *p* < 0.05 was considered statistically significant. All analyses were conducted using the SAS software (version 9.4 for Windows; SAS Institute, Cary, NC, USA).

## 5. Conclusions

The strong association between MMR gene variants and clinical outcomes reported in our study supported the role of DNA MMR deficiency in OSCC progression. *MSH2* rs3732183 and *MLH1* rs1800734 may serve as predictors of OSCC survival and may influence the response to adjuvant CCRT treatment, particularly in patients with advanced stages of OSCC. Our findings may require confirmation through additional studies with relatively large samples or those conducted on patients of other ethnicities.

## Figures and Tables

**Table 1:** Demographic and clinical characteristics of the patients with oral squamous cell carcinoma (OSCC) receiving adjuvant concurrent chemoradiotherapy.

Variable	Number (*n* = 319)	%
Mean age (SD), years	49.72 (9.8)	
Age, years (≥50)	155	48.59
Ethnicity		
Taiwanese	230	72.10
Hakka	72	22.57
Mainland Chinese	17	5.33
BMI, kg/m^2^		
<18.5	22	6.90
18.5–23.9	157	49.22
≥24	140	43.89
Smoking cigarettes (ever)	272	85.27
Drinking alcohol (ever)	221	69.28
Chewing betel quid (ever)	275	86.21
Drinking tea (ever)	156	48.90
Drinking coffee (ever)	76	23.82
Tumor differentiation *		
Well	51	16.14
Moderate	210	66.46
Poor	55	17.40
Primary tumor size		
T1–T2	122	38.24
T3–T4	197	61.76
Nodal involvement		
N0	38	11.91
N+	281	88.09
Perineural invasion (yes)	177	55.49
Vascular invasion (yes)	19	5.96
Lymphatic invasion (yes)	40	12.54
Extranodal extension (yes)	205	64.26
Pathological TNM Stage		
III	42	13.17
IV	277	86.83

BMI, body mass index; SD, standard deviation; TNM, tumor, nodes, and metastasis. * Tumor cell differentiation in 3 patients were not available.

**Table 2:** Univariate association of demographic and clinical factors with survival in patients with OSCC receiving adjuvant concurrent chemoradiotherapy.

Variable	No.	Event	OS		Event	DFS	
			HR (95% CI)	*p* Value		HR (95% CI)	*p* Value
Age, years							
<50	164	54	1.00		69	1.00	
≥50	155	40	0.71 (0.47–1.07)	0.104	60	0.82 (0.57–1.16)	0.262
Ethnicity							
Taiwanese	230	69	1.00		98	1.00	
Hakka	72	19	0.93 (0.56–1.56)	0.786	19	0.64 (0.39–1.04)	0.073
Mainland Chinese	17	6	1.25 (0.54–2.88)	0.601	12	1.80 (0.99–3.29)	0.056
BMI, kg/m^2^							
18.5–23.9	157	53	1.00		66	1.00	
<18.5	22	7	0.90 (0.41–1.97)	0.787	11	1.44 (0.76–2.72)	0.269
≥24	140	34	0.66 (0.43–1.02)	0.058	52	0.82 (0.57–1.20)	0.306
Smoking cigarettes							
Never	47	16	1.00		20	1.00	
Ever	272	78	0.89 (0.51–1.53)	0.661	109	0.96 (0.59–1.57)	0.874
Drinking alcohol							
Never	98	31	1.00		41	1.00	
Ever	221	63	1.00 (0.65–1.54)	0.994	88	1.00 (0.69–1.46)	1.000
Chewing betel quid							
Never	44	13	1.00		15	1.00	
Ever	275	81	0.96 (0.54–1.73)	0.900	114	1.37 (0.79–2.39)	0.268
Drinking tea							
Never	163	50	1.00		63	1.00	
Ever	156	44	0.82 (0.55–1.24)	0.359	66	1.06 (0.75–1.51)	0.745
Drinking coffee							
Never	243	78	1.00		93	1.00	
Ever	76	16	0.63 (0.37–1.07)	0.088	36	1.26 (0.85–1.86)	0.249
Tumor differentiation							
Well	51	19	1.00		24	1.00	
Moderate	210	61	0.92 (0.55–1.53)	0.735	80	0.89 (0.56–1.42)	0.622
Poor	55	13	0.81 (0.40–1.63)	0.550	24	1.14 (0.64–2.02)	0.662
Primary tumor size							
T1–T2	160	43	1.00		59	1.00	
T3–T4	159	51	1.41 (0.93–2.13)	0.102	70	1.47 (1.03–2.09)	0.034 *
Nodal involvement							
N0–N1	102	19	1.00		35	1.00	
N2–N3	217	75	2.38 (1.43–3.94)	0.0008 *	94	1.76 (1.17–2.63)	0.006 *
Perineural invasion							
No	142	41	1.00		56	1.00	
Yes	177	53	1.15 (0.77–1.74)	0.493	73	1.19 (0.83–1.70)	0.340
Vascular invasion							
No	300	86	1.00		122	1.00	
Yes	19	8	1.51 (0.73–3.13)	0.266	7	0.94 (0.44–2.02)	0.876
Lymphatic invasion							
No	279	75	1.00		110	1.00	
Yes	40	19	2.22 (1.32–3.72)	0.003 *	19	1.37 (0.82–2.30)	0.224
Extranodal extension							
No	114	14	1.00		36	1.00	
Yes	205	80	3.78 (2.14–6.68)	<0.0001 *	93	1.87 (1.26–2.78)	0.002 *
Pathologic TNM Stage							
III	42	10	1.00		14	1.00	
IV	277	84	1.62 (0.83–3.15)	0.154	115	1.66 (0.93–2.94)	0.086

BMI, body mass index; HR, hazard ratio; CI, confidence interval; OS, overall survival; DFS, disease-free survival. * Significance at *p* < 0.05.

**Table 3:** Univariate association between candidate single-nucleotide polymorphisms (SNPs) in mismatch repair (MMR) genes and survival in patients with OSCC receiving concurrent chemoradiotherapy.

Variable			No.	Event	OS			DFS	
					HR (95% CI)	*p* Value	Event	HR (95% CI)	*p* Value
MSH2									
	rs3732183 ^a^								
		AA	150	44	1.00		61	1.00	
		AG	125	36	0.94 (0.60–1.46)	0.779	54	1.02 (0.70–1.49)	0.913
		GG	34	8	0.64 (0.30–1.37)	0.253	8	0.47 (0.22–0.97)	0.042 *
		Additive model			0.85 (0.62–1.18)	0.337		0.81 (0.62–1.06)	0.120
		Dominant model	154	44	0.87 (0.57–1.32)	0.507	62	0.88 (0.61–1.26)	0.481
		Recessive model	34	8	0.66 (0.32–1.37)	0.268	8	0.46 (0.22–0.94)	0.034 *
		G-allele			0.84 (0.61–1.16)	0.290		0.79 (0.60–1.05)	0.102
MSH3									
	rs12515548 ^a^								
		CC	183	55	1.00		73	1.00	
		CT	122	35	0.93 (0.61–1.43)	0.742	49	1.02 (0.71–1.48)	0.909
		TT	13	3	0.83 (0.26–2.67)	0.760	6	1.44 (0.63–3.33)	0.391
		Additive model			0.93 (0.64–1.33)	0.676		1.09 (0.80–1.48)	0.590
		Dominant model	135	38	0.92 (0.61–1.40)	0.703	55	1.06 (0.74–1.51)	0.764
		Recessive model	13	3	0.86 (0.27–2.72)	0.795	6	1.43 (0.63–3.26)	0.395
		T-allele			0.93 (0.66–1.32)	0.691		1.08 (0.81–1.44)	0.610
	rs26279								
		AA	184	55	1.00		74	1.00	
		AG	122	36	0.97 (0.64–1.48)	0.885	49	1.02 (0.71–1.48)	0.906
		GG	13	3	0.78 (0.24–2.49)	0.671	6	1.30 (0.56–2.98)	0.544
		Additive model			0.94 (0.66–1.34)	0.729		1.07 (0.79–1.45)	0.675
		Dominant model	135	39	0.95 (0.63–1.44)	0.812	55	1.05 (0.73–1.49)	0.800
		Recessive model	13	3	0.79 (0.25–2.49)	0.683	6	1.28 (0.56–2.92)	0.552
		G-allele			0.94 (0.67–1.33)	0.741		1.06 (0.79–1.42)	0.689
EXO1									
	rs1047840 ^a^								
		GG	219	59	1.00		91	1.00	
		GA	93	31	1.34 (0.86–2.07)	0.198	33	0.85 (0.57–1.28)	0.438
		AA	6	3	2.84 (0.88–9.14)	0.081	4	2.68 (0.97–7.36)	0.056
		Additive model			1.43 (0.98–2.10)	0.067		1.02 (0.71–1.46)	0.934
		Dominant model	99	34	1.40 (0.92–2.15)	0.121	37	0.92 (0.62–1.36)	0.683
		Recessive model	6	3	2.59 (0.81–8.27)	0.108	4	2.80 (1.02–7.66)	0.045 *
		A-allele			1.39 (0.96–2.00)	0.078		1.02 (0.72–1.43)	0.937
MLH1									
	rs1800734								
		AA	100	36	1.00		44	1.00	
		AG	168	46	0.74 (0.47–1.14)	0.170	70	0.91 (0.62–1.33)	0.611
		GG	51	12	0.61 (0.32–1.17)	0.137	15	0.59 (0.33–1.06)	0.077
		Additive model			0.77 (0.56–1.04)	0.092		0.80 (0.62–1.04)	0.098
		Dominant model	219	58	0.70 (0.46–1.07)	0.101	85	0.82 (0.57–1.19)	0.302
		Recessive model	51	12	0.73 (0.40–1.34)	0.312	15	0.63 (0.36–1.07)	0.089
		G-allele			0.78 (0.58–1.05)	0.104		0.81 (0.63–1.05)	0.106

HR, hazard ratio; CI, confidence interval; OS, overall survival; DFS, disease-free survival. **^a^** Values is less than the total due to missing variables. * Significance at *p* < 0.05.

**Table 4:** Multivariate association between polymorphisms of MMR pathway genes and survival in patients with OSCC receiving adjuvant concurrent chemoradiotherapy.

Variable	OS		DFS	
	HR (95% CI)	*p* Value	HR (95% CI)	*p* Value
Age, years				
<50	1.00		1.00	
≥50	0.78 (0.51–1.19)	0.243	0.94 (0.65–1.36)	0.726
Ethnicity				
Taiwanese			1.00	
Hakka	-		0.68 (0.39–1.16)	0.158
Mainland Chinese	-		1.99 (1.04–3.82)	0.039 *
BMI kg/m^2^				
18.5–23.9	1.00			
<18.5	0.97 (0.44–2.17)	0.946	-	
≥24	0.73 (0.46–1.15)	0.175	-	
Drinking coffee				
Never	1.00			
Ever	0.67 (0.39–1.17)	0.157	-	
Primary tumor size				
T1–T2			1.00	
T3–T4	-		1.88 (1.19–2.98)	0.007 *
Nodular involvement				
N0–N1	1.00		1.00	
N2–N3	1.63 (0.96–2.79)	0.072	1.96 (1.04–3.69)	0.038 *
Lymphatic invasion				
No	1.00			
Yes	1.56 (0.91–2.66)	0.105	-	
Extranodal extension				
No	1.00		1.00	
Yes	2.91 (1.58–5.34)	0.0006 *	1.38 (0.87–2.20)	0.172
Pathologic TNM Stage				
III			1.00	
IV	-		0.66 (0.27–1.66)	0.380
MSH2 rs3732183				
AA			1.00	
AG	-		0.97 (0.65–1.44)	0.872
GG	-		0.45 (0.22–0.96)	0.039*
EXO1 rs1047840				
GG	1.00		1.00	
GA	1.16 (0.73–1.83)	0.532	0.83 (0.54–1.29)	0.411
AA	2.36 (0.72–7.80)	0.159	1.15 (0.34–3.88)	0.818
MLH1 rs1800734				
AA	1.00		1.00	
AG	0.67 (0.42–1.07)	0.091	0.74 (0.49–1.12)	0.158
GG	0.52 (0.27–1.01)	0.054	0.49 (0.26–0.92)	0.028 *

BMI, body mass index; HR, hazard ratio; CI, confidence interval, OS, overall survival; DFS, disease-free survival. * Significance at *p* < 0.05.

## Supplementary Materials

The following is available online at https://www.mdpi.com/2072-6694/11/5/598/s1, Figure S1: Linkage disequilibrium analysis between SNPs in *MSH3* (rs12515548 and rs26279).
