# Supplementary material for: Polymorphisms of Mismatch Repair Pathway Genes Predict Clinical Outcomes in Oral Squamous Cell Carcinoma Patients Receiving Adjuvant Concurrent Chemoradiotherapy
Source: Cancers (Basel). 2019 Apr 29;11(5):598. doi: 10.3390/cancers11050598 (PMC6562473; doi:10.3390/cancers11050598)
Supplement: Supplementary file 1 [file cancers-11-00598-s001.pdf]

Supplementary Material

Polymorphisms of Mismatch Repair Pathway Genes Predict Clinical Outcomes in Oral Squamous Cell Carcinoma Patients Receiving Adjuvant Concurrent Chemoradiotherapy

Thomas Senghore, Wen-Chang Wang, Huei-Tzu Chien, You-Xin Chen, Chi-Kuang Young, Shiang-Fu Huang and Chih-Ching Yeh

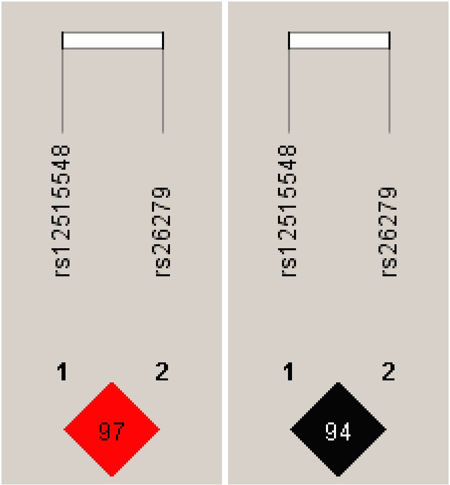

MSH3 (rs12515548 and rs26279)

Figure S1. Linkage disequilibrium analysis between SNPs in *MSH3* (rs12515548 and rs26279). The numbers in the squares indicate pairwise D′ (left) or R^2 (right) values and the corresponding shades of red or black represents the degree of linkage disequilibrium between pairs of SNPs.

© 2019 by the authors. Licensee MDPI, Basel, Switzerland. This article is an open access article distributed under the terms and conditions of the Creative Commons Attribution (CC BY) license (http://creativecommons.org/licenses/by/4.0/).
